# Resveratrol regulates insulin resistance to improve the glycolytic pathway by activating SIRT2 in PCOS granulosa cells

**DOI:** 10.3389/fnut.2022.1019562

**Published:** 2023-01-18

**Authors:** Aihong Liang, Wenmao Zhang, Qian Wang, Lan'e Huang, Jiaming Zhang, Duo Ma, Ke Liu, Shiyu Li, Xi Chen, Shan Li, Xiaocan Lei

**Affiliations:** ^1^Hunan Province Innovative Training Base for Medical Postgraduates, Hengyang Medical School, University of South China and Yueyang Women & Children's Medical Center, Hengyang, Yueyang, Hunan, China; ^2^Institute of Reproductive and Stem Cell Engineering, Central South University, Changsha, Hunan, China; ^3^Department of Reproductive Medicine, The First Affiliated Hospital, Hengyang Medical School, University of South China, Hengyang, Hunan, China

**Keywords:** resveratrol, PCOS, insulin resistance, SIRT2, glycolysis

## Abstract

**Scope:**

Insulin resistance (IR) has a close relationship with the main clinical manifestations of patients with PCOS; hence, the research and development of new drugs to treat PCOS by improving IR is a desiderate task at present. Resveratrol (RES) possesses a variety of beneficial pharmacological functions, such as antioxidation, anti-inflammatory, regulating glucose, and lipid metabolism. However, whether RES could improve IR and the underlying mechanisms remained unclear in PCOS.

**Methods and results:**

SD rats received a high-fat diet and letrozole for 30 days to establish the PCOS model and then intervened with RES for 30 days. The results demonstrated that RES played a protective role on the IR in PCOS rats, which significantly decreased the levels of blood glucose and serum insulin, up regulated the expression of IGF1R, and down regulated the expression of IGF1. *In vitro*, KGN cells were treated with insulin, RES, and AGK2, respectively. We found that a high dose of insulin (4μg/mL) significantly inhibited KGN cell viability, decreased the level of lactic acid, and increased the level of pyruvate, while RES (25μM) attenuated the growth-inhibitory effect, as well as increased the level of lactic acid and decreased the level of pyruvate after high levels of insulin treatment. Simultaneously, RES up regulated the expression level of the crucial rate-limiting enzymes relating to glycolytic pathways, such as LDHA, HK2, and PKM2. Furthermore, AGK2 remarkably inhibited the expression level of SIRT2, which was similar to the same negative effects processed by insulin. Meanwhile, RES overtly repaired the glycolysis process by reversing the levels of lactic acid and pyruvate, together with up regulating the expression level of LDHA, HK2, and PKM2, after AGK2 treatment.

**Conclusion:**

RES could effectively improve insulin resistance and restore the glycolysis pathway by regulating SIRT2, which may contribute to attenuating the ovarian damage of PCOS rats and provide a potential treatment for patients with PCOS.

## Introduction

Polycystic ovary syndrome (PCOS) is a common endocrine and metabolic disorder disease in women of childbearing age and affects up to 10%, characterized by hyperandrogenism, sparse ovulation, or anovulation (1). Insulin resistance (IR) and associated compensatory hyperinsulinemia are common features of PCOS affecting 50–70% of women with the disorder (2). IR has been defined as a decreased ability of insulin to regulate the uptake and production of glucose *in vivo*, which resulted in an increasing amount of insulin to achieve a given metabolic action (3). In the last few decades, numerous studies have identified the ovary as an important target tissue for insulin action (4). Physiological insulin levels promoted the proliferation of granulosa cells, and glucose metabolism of granulosa cells provided energy for the development of oocytes, thereby regulating follicular development (3). By contrast, hyperinsulinemia could promote the apoptosis of ovarian granulosa cells, increase the serum level of androgen, and aggravate hyperandrogenism in patients with PCOS, inhibiting the development of follicles (5–7). In addition, the literature studies have shown that IR could downregulate the expression of PI3K and inhibit PI3K/AKT pathway, reducing the function of insulin to regulate glucose metabolism, causing localized glucose metabolism disorder in the ovary (8, 9). These studies indicated that PCOS follicular dysplasia was closely related to hyperinsulinemia and IR. Meanwhile, multiple lines of evidence have reported that lactate, the main product of glycolysis, plays an important role during folliculogenesis and follicular maturation (10), and the rate of glycolysis was regulated by its rate-limiting enzymes, such as hexokinases (HKs), pyruvate kinase M (PKM), and lactic dehydrogenase (LDH) (11). Our previous research has indicated that abnormal glycolysis, in which the proteins' expression of HK2, PKM2, and LDHA were decreased, was a critical reason for follicular dysplasia in PCOS rats (12). However, the relationship between the IR and the abnormal glycolysis in the PCOS ovary remains ill-defined.

At present, vegetables and fruits rich in natural flavonoid compounds may prevent or delay the onset of diseases such as cardiovascular diseases (13). Resveratrol (3,4,5-trihydroxy-trans-stilbene, RES) is a naturally occurring compound mainly found in dietary plants including the root of polygonum cuspidate, peanut, and grape skin (14, 15). RES has several pharmacological effects including anti-inflammatory, antioxidant, and increased insulin sensitivity (14, 16). Hoseini et al. found that RES significantly decreased the level of insulin and fasting blood glucose in patients with type 2 diabetes complicated with coronary heart disease (17). Furthermore, Zou et al. have revealed that RES ameliorated metabolic disorders and IR in high-fat diet-fed mice (18). In addition, RES also has positive effects on follicular development, such as decreased number of atretic follicles, increased follicular reserve of the ovary, and prolonged ovarian lifespan (19, 20). Meanwhile, RES has a positive effect on improving the architecture of ovarian follicular (21) and suppressing the damage of the ovaries in PCOS rats by restoring glycolytic activity (12). However, the mechanism of RES on IR and the relationship between IR and glycolysis in PCOS remain unclear.

Thus, the present study aimed to examine the effect of RES on ovarian IR in PCOS rats, as well as to explore the mechanisms underlying the IR-regulated glycolysis pathway in PCOS ovarian granulosa cells, which may provide potential strategies for the treatment of PCOS.

## Materials and methods

### Animals and feeding

All experimental procedures were approved by the Animal Ethics Committee of the University of South China (permit number: USC2020031602). A total of 24 female Sprague-Dawley (SD) rats (5 weeks, 171 ± 11 g) were obtained from the Laboratory Animal Center of the University of South China (Hengyang, China; permit number: SYXK (Xiang) 2020-0002). The rats were raised with free access to food and water in a temperature-controlled room (23 ± 2°C) with a normal dark–light (12 h:12 h) cycle. After 7 days of acclimatization, rats were randomly divided into control (Ctrl) (*n* = 8) and PCOS (*n* = 16) groups. The control group received a normal diet, whereas the PCOS group received a high-fat diet (HFD) and letrozole to establish the PCOS model. The experimental group was then randomly divided into a PCOS group (*n* = 8) and a PCOS-RES group (*n* = 8), in which rats received oral RES (R8530; Solarbio, China).

### Dosage information

In the animal modeling experiment, rats were randomly divided into two groups. Ctrl group: rats were fed a normal diet for 30 days. PCOS group: rats were fed an HFD (consisting of 61.5% ordinary feed, 12% lard, 5% sucrose, 5% milk powder, 5% peanut, 10% egg, 1% sesame oil, and 0.5% salt) and intragastric administration of letrozole [1 mg/kg/d, dissolved in 1% carboxymethyl cellulose (CMC)] for 30 days (22, 23). During the treatment experiment, rats were divided into the Ctrl group, the PCOS group, and the PCOS-RES group. The Ctrl and PCOS rats were fed as described in the modeling experiment for 30 days. The PCOS-RES rats were fed a normal diet and intragastric administration of RES (20 mg/kg/d (24, 25), dissolved in 1% CMC) for 30 days.

### Determination of insulin levels

Blood was collected from the abdominal aorta of rats when killed and placed at room temperature (23 ± 2°C) for 30 min before centrifugation at 3,000 rpm for 15 min at 4°C. The level of serum insulin was detected using a radioimmunoassay (Beijing North Institute of Biological Technology, China).

### Cell culture and treatment

Human ovarian granulosa-like tumor cell line KGN, which originated from a stage III invasive ovarian granulosa cell carcinoma in a 63-year-old woman, was considered a model for understanding the regulation of steroidogenesis, cell growth, and apoptosis in human granulosa cells (26). In this study, KGN cells were kindly provided by Clinical Anatomy & Reproductive Medicine Application Institute. KGN cells were cultured with Dulbecco's Modified Eagle's Medium-high glucose (DMEM, Sigma, USA) supplemented with 12% fetal bovine serum (FBS, Invitrogen Gibco, USA) and maintained in an atmosphere of 5% CO_2_ at 37°C.

KGN cells were plated in 6-well plates at 10^5^ per well. After starving for 24 h, the cells were treated without or with insulin (I2643; Sigma, USA) at concentrations of 1, 2, and 4 mg/ml. Then, KGN cells were treated with insulin (4 μg/ml) and RES at concentrations of 10, 25, 50, and 100 μM for 24 h. In addition, the cells were also treated with AGK2 (10 μM, A8231; Sigma, USA) and RES (25 μM) for 24 h.

### CCK-8

KGN cells were plated in 96-well plates at 10^4^ per well. After starving for 24 h, the insulin group cells were treated without or with insulin at concentrations of 1, 2, and 4 μg/ml for 24 h. The KGN cells were treated with 4μg/ml of insulin, meanwhile treated with RES at concentrations of 0, 25, 50, and 100 μM for 24 h in the Ins + RES group, and the AGK2 group cells were treated with 10 μM, AGK2+RES were treated with 10 μM AGK2 and 25 μM resveratrol for 24 h. Then CCK-8 was added at 10 μl per well and incubated for 30 min in the incubator, following the manufacturer's protocol. Plates were read on a VersaMax microplate reader at 450 nm wavelength.

### Enzyme-Linked Immunosorbent Assay (ELISA)

Lactic acid and pyruvate in the culture supernatant were measured using commercially available ELISA kits. Plates were read on a VersaMax microplate reader at 505 nm (pyruvate) and 530 nm (lactic acid) wavelengths.

### Western blot

Ovarian tissues and cultured cells were homogenized in lysates [RIPA lysis buffer (CW2333S; CWBIO, China): PMSF (P0100; Solarbio, China) = 94:6] on ice for 30 min and subsequently centrifuged at 12,000 rpm for 20 min at 4°C. Supernatants were transferred to a clean 1.5 ml tube, and protein concentration was measured by BCA Protein Assay Kit (CW0014; CWBIO, China). Proteins were denatured by boiling at 100°C for 10 min and then separated by 10% SDS-PAGE, electrophoretically transferred onto polyvinylidene fluoride (PVDF) membranes, and blocked in PBST [phosphate-buffered saline (PBS) with 0.1% Tween-20] containing 5% skim milk powder for 2 h. After washing with PBST, the membranes were, respectively, incubated with antibodies against PKM2 (1:1,000, #4053; Cell Signaling Technology, USA), HK2 (1:1000, A0994; ABclonal, China), LDHA (1:1000, #3558; Cell Signaling Technology, USA), SIRT2 (1:1000, A12575; ABclonal, China), and GAPDH (1:1,000, #97166; Cell Signaling Technology, USA) overnight at 4°C. The membranes were further incubated with HRP-conjugated goat anti-mouse or goat anti-rabbit IgG (H+L) (1:5,000, SA00001-1/2; Protein Tech Group Inc., USA) for 2 h at room temperature. Finally, eECL (CW0049M, CWBIO, China) and the Tanon-5500 Chemiluminescence Imaging System were used to detect the chemiluminescence of protein bands.

### Quantitative real-time PCR (qPCR)

Total RNA from ovary tissues or KGN cells was extracted using the TRIzol reagent (15596018; Thermo Fisher Scientific, USA). The synthesis of cDNA was performed with the TransScript^®^ One-Step gDNA Removal and cDNA Synthesis SuperMix Kit (AT311-02; TransGen Biotech, China), according to the manufacturer's protocol. Real-time PCR analyses were performed with ChamQ Universal SYBR qPCR Master Mix (Q711-02; Vazyme, China) and Applied Biosystems QuantStudio 3 (Thermo Fisher Scientific, USA). GAPDH was used as the reference, and gene expression levels were calculated using the comparative Ct method (27). The primer sequences used for amplification are shown in Table 1.

**Table 1:** Primer sequences used for the qRT-PCR analysis.

**GENE**	**Sequence (5^′^-3^′^)**	**Annealing temperature (°C)**	**Product (bp)**
Rat IGF1	F: GTGGTGAATGACACAGTTGG	55	164
	R: CACATTACGCATCTCTTCCA		
Rat IGF1R	F: ACATCCTGTGGCTGGACTAT	55	179
	R: TCCACTTCTGTCACCAGGTA		
	R: GCAGATGGTCGGCTTGAAC		
Rat GAPDH	F: CCTCAAGATTGTCAGCAATG	55	164
	R: CAGTCTTCTGAGTGGCAGTG		
Homo HK2	F: CGAGAGCATCCTCCTCAAGTG	55	164
	R: AGCCACAGGTCATCATAGTTCC		
Homo PKM2	F: TGGGAGAGAAGGGAAAGAACATC	55	179
	R: GCACCGTCCAATCATCATCTTC		
Homo LDHA	F: ATGAGTTGGACTGTGCCTGTTGTG	55	134
	R: GTGAAGAGCCAGGTGCCGTTG		
Homo SIRT2	F: CGCACGGCACCTTCTACACATC	55	188
	R: GGCTCTGACAGTCTTCACACTTGG		
Homo GAPDH	F: GAGTCCACTGGCGTCTTCAC	55	164
	R: GAGGCATTGCTGATGATCTTGAG		

### Statistical analysis

Data were analyzed using GraphPad Prism 8.0 (GraphPad Software, USA) and presented as the mean ± standard deviation. Significant differences within groups were evaluated by the unpaired one-way ANOVA followed by Bonferroni's *post-hoc* test.

## Results

### Resveratrol enhanced the ovarian insulin sensitivity in PCOS rats

**Figure 1:**
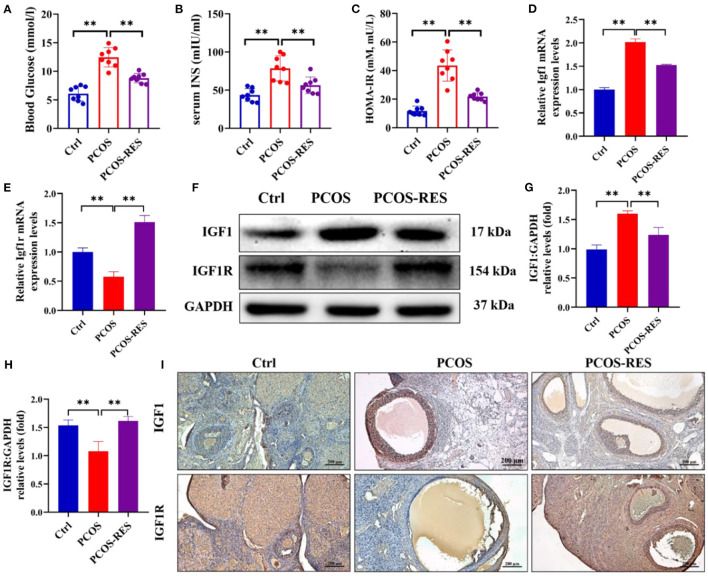
Resveratrol can enhance the ovarian insulin sensitivity in PCOS rats. The level of blood glucose **(A)**, serum insulin **(B)**, and HOMA-IR **(C)** was shown (*n* = 8). The relative mRNA expression levels of IGF1 **(D)** and IGF1R **(E)** in the ovary were determined using qPCR. The protein levels of IGF1 and IGF1R were determined by Western blot **(F)** and quantified by Image J software [IGF1 **(G)** and IGF1R **(H)**]. Immunohistochemistry analysis of the expression of IGF1 and IGF1R **(I)**. Significant differences between the groups are shown as **P < 0.01.

A PCOS rat model was first established to examine the effects of RES on ovarian insulin sensitivity. As shown in Figures 1A–C, HFD and letrozole treatment remarkably increased the levels of blood glucose, serum insulin, and HOMA-IR compared with that of the Ctrl group (*n* = 8 each; *p* < 0.01). The levels of blood glucose and serum insulin were obtained after rats were exposed to PCOS-inducing conditions for 30 days. RES administration significantly reduced the levels of blood glucose, serum insulin, and HOMA-IR of PCOS rats (*n* = 8 each; *p* < 0.01) (Figures 1A–C). To explore the effect of RES on ovarian insulin resistance, we detected the expressions of IGF1 and IGF1R. The results of qPCR showed the upregulation of IGF1 and downregulation of IGF1R in PCOS rats, and RES administration significantly restored the mRNA expression of IGF1 and IGF1R (Figures 1D, E). Western blot analysis confirmed the increase of IGF1 and decrease of IGF1R in the PCOS group vs. the Ctrl group, respectively, and the improvement in response to RES treatment (Figures 1F–H). Immunohistochemistry (IHC) analysis confirmed the results, showing an increase of IGF1 and a decrease of IGF1R in the PCOS group and a recovery of values in response to RES treatment (Figure 1I).

### Resveratrol improved the glycolysis process of insulin-treated KNG cells

**Figure 2:**
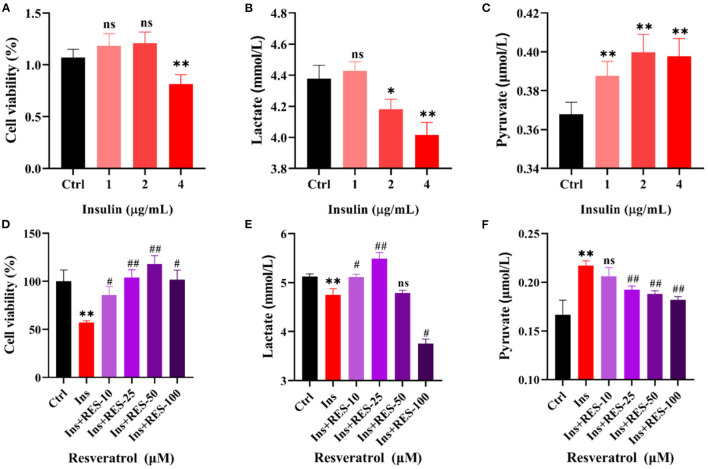
RES can improve the glycolysis process of insulin-treated KGN cells. Cell viability of KGN cells in which treatment with insulin **(A)** or insulin and RES **(D)** was determined using cck-8. The level of lactate in KGN cells, which treated with insulin **(B)** or insulin and RES **(E)** using ELISA. The level of pyruvate in KGN cells, which treated with insulin **(C)** or insulin and RES **(F)** using ELISA. *ctrl vs. insulin; ^#^insulin vs. insulin + RES. Significant differences between the groups are shown as *P < 0.05, **P < 0.01, ^#^*P* < 0.05, and ^##^*P* < 0.01.

Then, we deeply explored the mechanism of RES enhancing insulin sensitivity at the cellular level. As shown in Figures 2A–C, we treated KGN cells with different concentrations of insulin and detected the changes in cell viability, and the levels of lactic acid and pyruvic acid. The results of CCK-8 showed 4 μg/ml of insulin decreased KGN cell viability compared with that of the Ctrl group, while there was no significant difference in KGN cells after being treated with 1 and 2 μg/ml of insulin. The results of ELISA showed that with the increase in the concentration of insulin, the level of lactate decreased while pyruvate increased. Thus, we choose the 4 μg/ml insulin for the next experiment. With respect to the concentration of insulin, we added different concentrations of RES to cells. The results showed that 25 and 50 μm of RES could reduce the pyruvate level and increase the lactate level of cells treated with insulin (Figures 2D–F). Lactate has long been proposed to play an important role in follicular development (10); thus, we chose 25 μm of RES, which is the most significant in increasing lactate level for the follow-up experiments.

### Resveratrol improved the expression of key glycolytic enzymes of insulin-treated KNG cells

**Figure 3:**
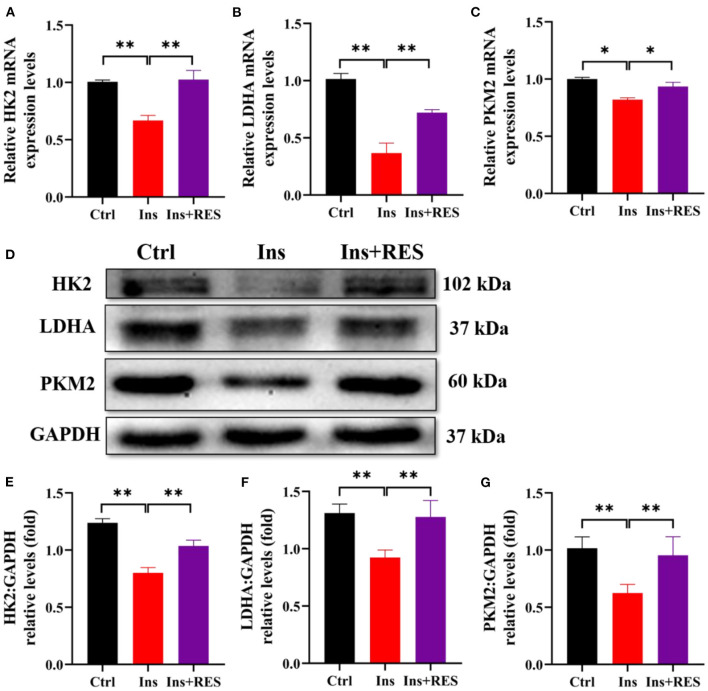
RES can improve the expression of key glycolytic enzymes of insulin-treated KGN cells. The relative mRNA expression levels of HK2 **(A)**, LDHA **(B)**, and PKM2 **(C)** in KGN cells were determined using qPCR. The protein levels of HK2, LDHA, and PKM2 were determined by Western blot **(D)** and quantified by ImageJ software **(E–G)**. Significant differences between the groups are shown as *P < 0.05 and **P < 0.01.

The aforementioned experimental results showed that the intervention of RES could enhance the insulin sensitivity of ovarian tissue and promote the process of glycolysis. To explore the effect of insulin resistance on glycolysis, we detected the expression of key glycolytic enzymes (HK2, PKM2, and LDHA) in KGN cells after insulin treatment and RES intervention. The results of qPCR showed the mRNA expressions of HK2, LDHA, and PKM2 were downregulated in KGN cells treated with insulin. RES administration significantly restored the mRNA expression of HK2, LDHA, and PKM2 (Figures 3A–C). Western blot analysis confirmed that insulin decreased the protein expressions of HK2, LDHA, and PKM2 in KGN cells compared with the Ctrl group and the improvement after RES treatment (Figures 3D–G).

### Resveratrol upregulated the expression of SIRT2 in KGN cells treated with insulin and AGK2

**Figure 4:**
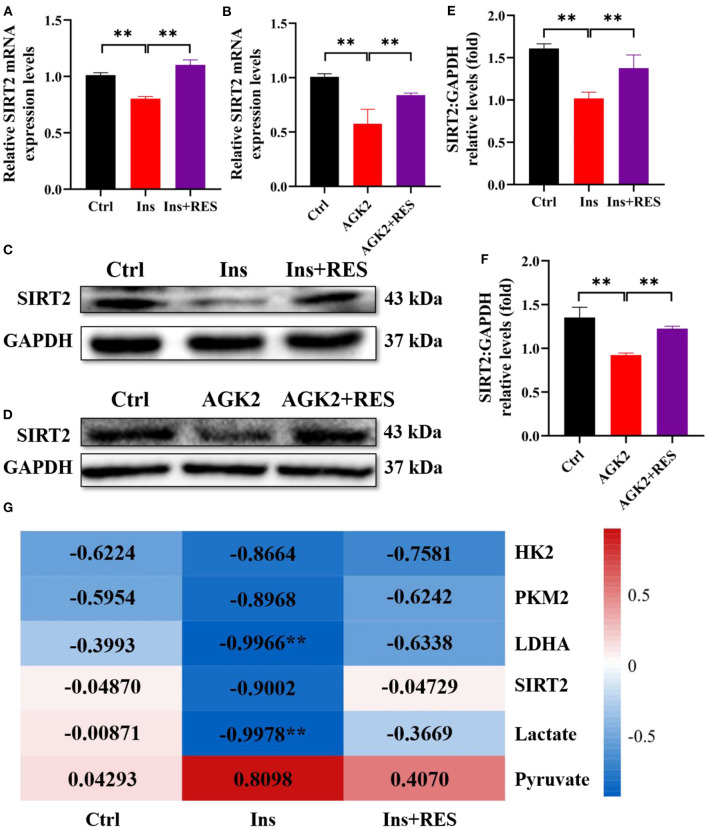
RES can upregulate the expression of SIRT2 in KGN cells treated with insulin and AGK2. The relative mRNA expression levels of SIRT2 **(A, B)** in KGN cells were determined using qPCR. The protein level of SIRT2 was determined by Western blot **(C, D)** and quantified by ImageJ software **(E, F)**. Correlation analysis between insulin sensitivity and glycolysis **(G)**. Significant differences between groups are shown as **P < 0.01.

Based on the transcriptome profiling and ovary tissue results in our previous research, we found that SIRT2, an NAD^+^-dependent deacetylase, was decreased in PCOS rats while increased in PCOS-RES rats (12). In this study, we mainly interrogated the role of SIRT2 in PCOS granulosa cells. First, we detected the mRNA level of SIRT2 in KGN cells treated with insulin and RES, and the results showed that RES could reverse the effect of insulin on SIRT2 (Figure 4A). AGK2 is a specific SIRT2 inhibitor, which is reported to confer neuroprotection (28, 29). To future confirm the effect of RES, we added AGK2 in KGN cells and found that the expression of SIRT2 was significantly downregulated after being treated with AGK2. As expected, RES reversed it (Figure 4B). Meanwhile, Western blot analysis confirmed that the protein expression of SIRT2 was decreased in KGN cells treated with insulin compared with the Ctrl group and increased after RES treatment (Figures 4C–F). To explore the correlation between insulin sensitivity and the glycolysis process in PCOS, we analyzed the correlation between HOMA-IR and key rate-limiting enzymes (HK2, LDHA, and PKM2), glycolytic pathway regulatory factor (SIRT2) and glycolysis products (lactic acid and pyruvate). The results showed that HOMA-IR was negatively correlated with HK2, LDHA, PKM2, SIRT2, and lactic acid levels and had little correlation after RES intervention (Figure 4G), which indicated that RES intervention promoted glycolysis by improving ovarian insulin resistance in PCOS rats.

### Resveratrol reversed the inhibitory effect of AGK2 on the process of glycolysis in KGN cells

**Figure 5:**
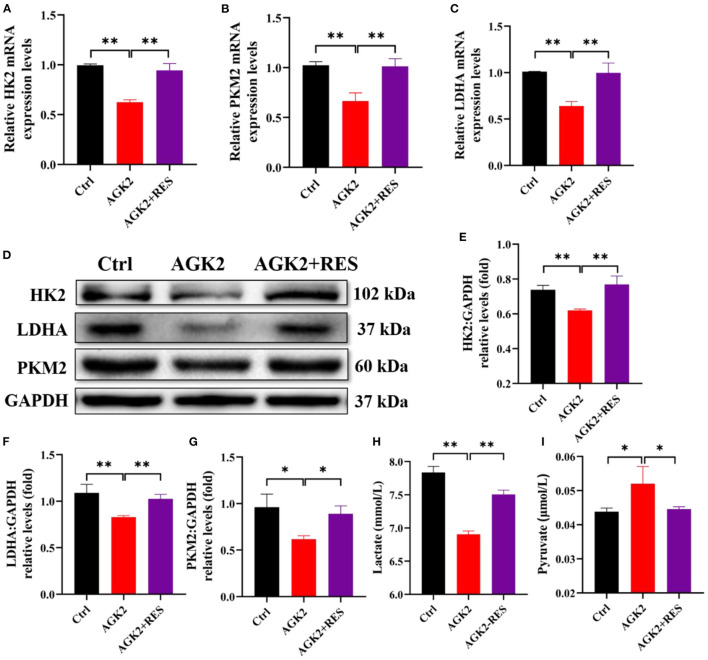
RES can upregulate the expression of SIRT2 in KGN cells treated with insulin and AGK2. The relative mRNA expression levels of HK2 **(A)**, PKM2 **(B)**, and LDHA **(C)** in KGN cells were determined using qPCR. The protein levels of HK2, LDHA, and PKM2 were determined by Western blot **(D)** and quantified by ImageJ software **(E–G)**. The levels of lactate and pyruvate in KGN cells using ELISA **(H, I)**. Significant differences between the groups are shown as *P < 0.05 and **P < 0.01.

To find out whether RES regulates glycolysis of granulosa cells through SIRT2, we used qPCR and Western blot to detect the mRNA levels and protein expressions of HK2, PKM2, and LDHA in each group. The results showed that compared with the Ctrl group, the mRNA levels and protein expressions of HK2, PKM2, and LDHA were significantly decreased in KGN cells with AGK2 treatment while significantly increased after RES intervention (Figures 5A–C). Western blot analysis confirmed that the expression of SIRT2 in KGN cells treated with AGK2 was significantly decreased than the Ctrl group and increased after RES treatment (Figures 5D–G). Meanwhile, we detected the levels of lactic acid and pyruvic acid in each group of cells by ELISA to further verify the regulation of SIRT2 on the glycolysis activity of granulosa cells. The results showed that compared with the Ctrl group, AGK2 could significantly reduce the content of lactic acid and increase the accumulation of pyruvate, which significantly improved after RES intervention (Figures 5H, I). This is consistent with the changes in PCOS-like cells constructed by insulin and the levels of glycolysis products in PCOS rats, which further proves that SIRT2 played a key role in RES enhancing insulin sensitivity, improving the glycolysis process of granulosa cells in PCOS rats.

## Discussion

Recently, the increase in the incidence of PCOS has affected the physical and mental health of women of childbearing age, even as young girls (30). RES is under consideration as a new agent for the treatment of PCOS symptoms (21). Our previous study first explored that disorder glycolysis plays an important role in reproductive disorders of PCOS, and RES improves the PCOS rats' follicular development by regulating the glycolytic pathway (12). However, the underlying mechanism of the RES-regulated glycolytic pathway to reverse the reproductive disorders in PCOS is still unclear. Thus, in this study, we investigated the molecular mechanism by which RES improved the follicular development of PCOS. Reports showed that insulin resistance associated with compensatory hyperinsulinemia is a common feature of PCOS affecting 50–70% of women along with ovulation failure (23). Therefore, in this study, we determined the insulin resistance in PCOS rats, the results showed higher levels of glucose and insulin in PCOS rats, and HOMA-IR also increased. Those results were consistent with the study reported by Peng et al. (23), which lays a foundation for subsequent experiments.

A follicle is the basic functional unit of the mammalian ovary, which is structurally divided into oocytes, granulosa cells, and theca cells (31). Studies have shown that IGF is involved in many physiological processes such as follicular growth, steroid synthesis and secretion, follicular atresia, and oocyte maturation, and its expression changes are closely related to insulin resistance (32). It was found that the serum IGF1 level and insulin resistance index (HOMA-IR) of patients with PCOS were significantly increased, and IGF1 was positively correlated with HOMA-IR (33). Similarly, this study found that the expression of IGF1 in the ovarian tissue of PCOS rats was positively correlated with HOMA-IR. Studies have shown that lowering the level of IGF1 effectively improved the serum level of testosterone in PCOS rats and reduced the pathological damage of ovarian tissue (34). The results of this study proved that RES also reduced the expression of IGF1 in ovaries and enhanced insulin sensitivity of ovaries in PCOS rats, which provide a theoretical basis for RES to treat patients with PCOS.

Granulosa cells are the most important somatic cells in the process of follicular development, the change in their morphology and the number is the starting signal of follicular growth and development. As the oocytes lack the glycolytic rate-limiting enzymes, their ability to utilize glucose is rarely low, while granulosa cells have a strong affinity for glucose, transforming glucose into lactic acid, pyruvate, and other substrates, which are essential substances for oocyte growth and development (35, 36). Therefore, the glycolysis rate of granulosa cells is of great significance to the development of follicles and oocytes. Studies have found that the expression of key rate-limiting enzymes of glycolysis: LDHA, HK2, and PKM2 in ovaries of PCOS mice was significantly lower than that of normal mice, and lactic acid production was reduced (37, 38). However, increasing the content of lactic acid in the follicular fluid of patients with PCOS *in vitro* reduced the occurrence of atresia follicles (39). Lin et al.'s research on the follicular fluid of normal subjects and patients with PCOS showed that the proper amount of insulin could promote the production of lactic acid in the follicular fluid of normal subjects but has no significant effect on the content of lactic acid in the follicular fluid of patients with PCOS (40). *In vitro* experiments of this subject found that a high dose of insulin inhibited the activity of human ovarian granulosa cells (KGN cells) and reduced the lactic acid content in cells. Similarly, Yan et al. found that insulin resistance could reduce the expression of HK2 in neurons, resulting in the blockage of glycolysis, which confirmed that high levels of insulin could inhibit glycolysis (41). This study confirmed that the HOMA-IR of PCOS rats was negatively correlated with the rate of ovarian glycolysis, and the correlation decreased after the intervention of RES, which further clarified the mechanism of RES in improving follicular development disorder in PCOS rats by enhancing insulin sensitivity and promoting the glycolysis process of granulosa cells.

Studies have shown that RES plays an anti-inflammatory and antioxidant role by activating the expression of Sirtuins in tissues or cells (42). Sirtuins are an NAD^+^-dependent deacetylase expressed in the whole life system, including SIRT1-7, and SIRT2 mainly exists in the cytoplasm and nucleus, takes key enzymes of glycolysis as targets, and participates in regulating lipid metabolism, glucose metabolism, and oxidative stress (43). Lantier et al. found that knocking out SIRT2 could reduce muscle sensitivity and promote insulin resistance in the liver (44). The results of this study showed that a high dose of insulin *in vitro* could downregulate the expression of SIRT2 in KGN cells, which indicated that the expression of SIRT2 is also affected by insulin level. In addition, SIRT2 also participated in the regulation of cell glycolysis. Knocking out SIRT2 in an islet could destroy the stability of glucokinase regulatory protein and reduce glycolysis flux (45). AGK2 is a selective SIRT2 inhibitor with an IC50 value of 3.5 μM. Previous studies showed that the addition of AGK2 inhibited SIRT2 activity and rescued the α-Synuclein-mediated toxicity of dorsomedial dopamine neurons (46). Meanwhile, in SIRT2-myc-expressing HeLa cells, AGK2 effectively inhibits the activity of SIRT2 and increases acetylated tubulin (29). *In vitro* experiments of this subject also proved that SIRT2 activity was inhibited by AGK2 and RES reversed it.

In summary, this topic elaborated the mechanism that RES could enhance the insulin sensitivity of the ovary, promote the glycolysis activity of ovarian granulosa cells, and improve the follicular development disorder of PCOS rats from the animal level and cell level. At the same time, SIRT2 may be the key factor for RES to regulate the rate of glycolysis in ovarian granulosa cells. The research results of this topic further improve the mechanism of PCOS follicular development disorder and provide a new direction and strategy for the clinical prevention and treatment of PCOS diseases.

## Data availability statement

The original contributions presented in the study are included in the article/supplementary material, further inquiries can be directed to the corresponding authors.

## Ethics statement

The animal study was reviewed and approved by Animal Ethics Committee of the University of South China (permit number: USC2020031602).

## Author contributions

XL: conceptualization. AL, LH, JZ, DM, KL, and SL: methodology. XL and ShaL: validation. AL, LH, WZ, QW, XC, SL, and JZ: formal analysis, investigation, and data curation. AL: writing—original draft preparation. AL and XL: writing—reviewing and editing. All authors contributed to the article and approved the submitted version.
